# Molecular mechanism of chemoresistance by miR-215 in osteosarcoma and colon cancer cells

**DOI:** 10.1186/1476-4598-9-96

**Published:** 2010-04-30

**Authors:** Bo Song, Yuan Wang, Matthew A Titmus, Galina Botchkina, Andrea Formentini, Marko Kornmann, Jingfang Ju

**Affiliations:** 1 Translational Research Laboratory, Department of Pathology, School of Medicine, Stony Brook University, Stony Brook, New York 11794, USA; 2 Department of Surgery, School of Medicine, Stony Brook University, Stony Brook, New York 11794, USA; 3 Basic Medical Sciences, Wuhan University, Wuhan 430072, China; 4 Department of General, Visceral and Transplantation Surgery, University of Ulm, Ulm 89075, Germany

## Abstract

**Background:**

Translational control mediated by non-coding microRNAs (miRNAs) plays a key role in the mechanism of cellular resistance to anti-cancer drug treatment. Dihydrofolate reductase (DHFR) and thymidylate synthase (TYMS, TS) are two of the most important targets for antifolate- and fluoropyrimidine-based chemotherapies in the past 50 years. In this study, we investigated the roles of miR-215 in the chemoresistance to DHFR inhibitor methotrexate (MTX) and TS inhibitor Tomudex (TDX).

**Results:**

The protein levels of both DHFR and TS were suppressed by miR-215 without the alteration of the target mRNA transcript levels. Interestingly, despite the down-regulation of DHFR and TS proteins, ectopic expression of miR-215 resulted in a decreased sensitivity to MTX and TDX. Paradoxically, gene-specific small-interfering RNAs (siRNAs) against DHFR or TS had the opposite effect, increasing sensitivity to MTX and TDX. Further studies revealed that over-expression of miR-215 inhibited cell proliferation and triggered cell cycle arrest at G2 phase, and that this effect was accompanied by a p53-dependent up-regulation of p21. The inhibitory effect on cell proliferation was more pronounced in cell lines containing wild-type p53, but was not seen in cells transfected with siRNAs against DHFR or TS. Moreover, denticleless protein homolog (DTL), a cell cycle-regulated nuclear and centrosome protein, was confirmed to be one of the critical targets of miR-215, and knock-down of DTL by siRNA resulted in enhanced G2-arrest, p53 and p21 induction, and reduced cell proliferation. Additionally, cells subjected to siRNA against DTL exhibited increased chemoresistance to MTX and TDX. Endogenous miR-215 was elevated about 3-fold in CD133+HI/CD44+HI colon cancer stem cells that exhibit slow proliferating rate and chemoresistance compared to control bulk CD133+/CD44+ colon cancer cells.

**Conclusions:**

Taken together, our results indicate that miR-215, through the suppression of DTL expression, induces a decreased cell proliferation by causing G2-arrest, thereby leading to an increase in chemoresistance to MTX and TDX. The findings of this study suggest that miR-215 may play a significant role in the mechanism of tumor chemoresistance and it may have a unique potential as a novel biomarker candidate.

## Background

Antifolate- and fluoropyrimindine-based chemotherapies are widely used to reduce the recurrence rates and improve the survival of a number of tumors, including osteosarcoma and colorectal cancer. However, resistance to chemotherapeutic agents is still one of the major reasons for the failure of cancer treatment. Previous efforts have mainly focused on the relationship between the target levels of dihydrofolate reductase (DHFR) or thymidylate synthase (TYMS, TS) and their response to inhibitors such as methotrexate (MTX) and Tomudex (TDX). One of the important chemotherapeutic targets for antifolate-based chemotherapy, DHFR, catalyzes the reduction of dihydrofolate to tetrahydrofolate as the one-carbon donor essential for the *de novo *synthesis of thymidylate (dTMP), a precursor for DNA biosynthesis [1]. TS catalyzes the reductive methylation of dUMP to dTMP [2]. Due to their critical functions, both DHFR and TS have been the major anti-cancer targets for the past 50 years. However, it still remains a debated issue whether DHFR or TS can be used as predictive or prognostic biomarkers [3-6]. Clearly, the time has come to move beyond discussions of target/drug relationships, and broaden our search to include novel biomarkers that would allow us to better relate clinical response to chemotherapy.

It has been well documented that post-transcriptional and translational controls play a key role in the mechanism of cellular resistance to anti-cancer drug treatment [7-12]. One relatively newly-identified mechanism of translational control is mediated by small, non-coding single-stranded RNAs termed microRNAs (miRNAs). miRNAs are complementary to and regulate the translation of one or more mRNA molecules, most likely by binding to their 3'UTRs and inhibiting mRNA translation or facilitating mRNA cleavage in mammalian cells [13], although many of the mechanistic details are yet to be elucidated [14,15]. Furthermore, a given species of miRNA can perfectly or imperfectly base pair with multiple targets, allowing it to potentially regulate the translation of numerous mRNAs. It has been predicted that over 30% of the human protein coding genes are post-transcriptionally regulated by this mechanism [16-18]. Given the major roles of miRNAs in the regulation of protein expression in general, it is crucial to understand the contributions of miRNAs to tumor chemoresistance.

Previous study from our laboratory has shown a number of miRNAs may be directly regulated by tumor suppressor gene p53 [19], and this novel mechanism has been proved to be critical as part of the p53 function in regulating cell cycle and proliferation [20-24]. Recently, we have also confirmed that the expression of miR-192 is directly regulated by p53 and that one of the major targets of miR-192 is DHFR [25]. To date no miRNAs have been reported to target TS, whose expression is known to be regulated at transcriptional and post-transcriptional levels.

In this study, we described a novel mechanism of chemoresistance mediated by miR-215. We provided experimental evidence that although miR-215 reduced the expression of both DHFR and TS, the over-expression of miR-215 also counter-intuitively decreased chemosensitivity to the DHFR inhibitor MTX and the TS inhibitor TDX. In contrast, small-interfering RNAs (siRNAs) mediated knock-down of DHFR or TS increased cellular sensitivity to MTX or TDX, respectively. This difference was likely due, at least to a large degree, to reduced cell proliferation rate and cell cycle G2-arrest mediated by miR-215 through down-regulation of denticleless protein homolog (DTL) and increased p53 and p21. DTL, also known as retinoic acid-regulated nuclear matrix-associated protein (RAMP), or DNA replication factor 2 (CDT2), is reported to be correlated with the cell proliferation, cell cycle arrest and cell invasion in hepatocellular carcinoma, breast cancer and gastric cancer [26-28]. Furthermore, the expression of miR-215 was elevated in CD133+HI/CD44+HI human colon cancer stem cells, leading to their slow proliferation rate and allowing them to resist the damage caused by chemotherapeutic agents [29]. Inversely, the expression of miR-215 was significantly decreased in colorectal tumor specimens compared to adjacent normal colorectal tissues, contributing to the fast-proliferating, chemotherapy-sensitive phenotype of differentiated cancer cells. As a result, miR-215 may be a potential important target for developing novel anti-cancer therapeutics and a biomarker candidate in tumor chemoresistance.

## Methods

### Cell culture and reagents

The human osteosarcoma cell lines U-2 OS, MG63 were obtained from the American Type Culture Collection (ATCC). The human colon cancer cell lines HCT 116 (wt-p53) and HCT 116 (null-p53) were a gift from Professor Bert Vogelstein (The Johns Hopkins University). U-2 OS, HCT 116 (wt-p53) and HCT 116 (null-p53) cells were maintained in McCoy's 5A medium (Gibco Laboratories), and MG63 cells were maintained in Eagle's Minimum Essential Medium (ATCC) respectively. All the media were supplemented with 10% dialyzed fetal bovine serum (HyClone Laboratories). MTX, TDX, cisplatin and doxorubicin were purchased from Sigma-Aldrich.

### Patients and samples

Microscopically confirmed tumor samples and paired adjacent normal tissues were obtained from 24 patients undergoing surgical resection of primary colorectal adenocarcinoma at the Department of General, Visceral and Transplantation Surgery, University of Ulm, Germany. Following surgery, samples from tumor and adjacent normal tissues were frozen in liquid nitrogen and stored at -80°C for subsequent RNA extraction. Patient consent forms were obtained from all patients according to the institutional regulations. The characteristics of these patients are shown in Additional file 1.

### Isolation of colon cancer stem cells

HCT 116 (wt-p53) cells were sorted with multiparametric flow cytometry using BD FACS Aria cell sorter (Becton Dickinson) under sterile conditions. Cells were prepared and labeled with conjugated anti-human CD133-PE (clone 105902; R&D Systems) and CD44-FITC (clone F10-44-2; R&D Systems). Antibodies were diluted in MACS buffer containing 5% BSA, 1 mM EDTA and 15-20% blocking reagent (Miltenyi Biotec) to inhibit non-specific binding to non-target cells. After 15 min incubation at 4°C, staining cells were washed, resuspended in 500 μl of MACS buffer, and sorted.

### Transfections of miR-215 and siRNAs specific for DHFR, TS or DTL

U-2 OS, MG63, HCT 116 (wt-p53) and HCT 116 (null-p53) cells (2 × 10^5^) were plated in six-well plates and transfected with 100 nM of either miR-215 mimics or non-specific miRNA (Ambion) after 24 h by Oligofectamine (Invitrogen) according to the manufacturer's protocols. siRNA against DHFR (ON-TARGET plus SMARTpool L-008799-00-0010, human DHFR, NM_000791), siRNA against TS [30], and siRNA against DTL (ON-TARGET plus SMARTpool L-020543-00-0005, human DTL, NM_016448) were purchased from Dharmacon and transfected with Oligofectamine at a final concentration of 100 nM.

### miR-215 knock-down

To knock down endogenous miR-215, HCT 116 (wt-p53) cells were transfected with 100 nM of scramble-miR locked nucleic acid (LNA-control) or LNA anti-miR215 (LNA-miR215) oligonucleotides by Lipofectamine 2000 (Invitrogen) in six-well plates (2 × 10^5 ^cells/well). LNA-control and LNA-miR215 were purchased from Exiqon. To mimic the stress situation, HCT 116 (wt-p53) and HCT 116 (null-p53) cells were first transfected with 100 nM of miR-215 in six-well plates (2 × 10^5 ^cells/well), 24 h later, 100 nM of LNA-control or LNA-miR215 were transfected into the cells respectively.

### RNA isolation

Total RNAs, including miRNAs, were isolated using TRIzol reagent (Invitrogen) according to the manufacturer's instructions (see Additional file 2).

### Real time qRT-PCR analysis of miR-215

The relative quantity of miR-215 was evaluated by real time qRT-PCR (details see Additional file 2).

### Real time qRT-PCR analysis of mRNA expression

The levels of DHFR and TS mRNAs were determined as described in Additional file 2.

### Cell proliferation and cell cycle analyses

Cell proliferation and cell cycle analyses were performed, for details see Additional file 2.

### Western immunoblot analysis and antibodies

Western immunoblot was performed, details and information for antibodies see Additional file 2.

### Plasmid construction, transfection and luciferase assays

pMIR-REPORT Luciferase miRNA Expression Reporter Vector (Ambion) was used to determine the targets of miR-215. Double stranded DNA oligonucleotides containing the miR-215 binding sequence (wild-type, underlined) or a mismatch sequence (mutant, italic) of the 3'UTR of DHFR or TS mRNAs and the HindIII and SpeI restriction site overhangs were synthesized (IDT). After annealing, double strand oligonucleotides were inserted into the pMIR-REPORT plasmid, downstream of the firefly luciferase reporter. The sequences of these synthesized oligonucleotides are listed in Additional file 3. The 3'UTR of TS includes 2 binding sites of miR-215 at 84-104 bp (wild type-1) and 216-236 bp (wild type-2) respectively. Transfection and luciferase assays were performed as described in Additional file 2.

### MTX and TDX chemosensitivity

U-2 OS and HCT 116 (wt-p53) cells were re-plated in 96-well plates at 2 × 10^3 ^cells/well in triplicate after being transfected with miR-215 mimics, non-specific miRNA, or siRNAs against DHFR, TS or DTL in 100 μl of medium. After twenty-four hours, 10-200 nM of MTX or TDX in 100 μl medium was added, and the cells were incubated for another 72 h. WST-1 was added to each well (10 μl), and after 2 h incubation, optical absorbance was measured at 450 and 630 nm respectively. HCT 116 (wt-p53) cells were transfected with LNA antisense miRNAs and assayed for MTX sensitivity using the same methods described above.

### Statistical analysis

All experiments were repeated at least twice. Statistical significance between the two groups of data was evaluated by Student's *t *test (two-tailed). Asterisks indicate significant differences of experimental groups compared with the corresponding control condition. Statistical analysis was performed using GraphPad Prism software 5 (GraphPad, Inc.). The expression Δ*C*_T _value of miR-215 in each clinical sample was calculated by normalizing with its internal control RNU6B and relative quantitation values were plotted using SDS software v1.2 (Applied Biosystems). The statistically significant difference in expression level between tumor and normal tissues was calculated using a paired Wilcoxon signed-rank test, and the statistical analysis was performed by MedCalc^® ^10.0.2 (MedCalc software, Belgium). Differences were considered statistically significant at *P *< 0.05.

## Results and Discussion

The nature of the relationship, DHFR or TS expression and their response to the inhibitors MTX or TDX has been long debated [31]. More broadly, it is also becoming increasingly clear that progress in the area of cancer treatment necessitates that we move beyond the target/drug relationship and begin to consider more seriously the value of biomarkers in the evaluation of clinical response to treatment. In this study, rather than focusing only on the interactions of DHFR or TS levels, we investigated the mechanisms of chemoresistance from a new angle by considering the roles played by miRNAs. Our findings suggest that the influences of miR-215 on chemosensitivity to MTX or TDX are more important than the target levels (DHFR, TS) alone.

### DHFR and TS are direct targets of miR-215

**Figure 1:**
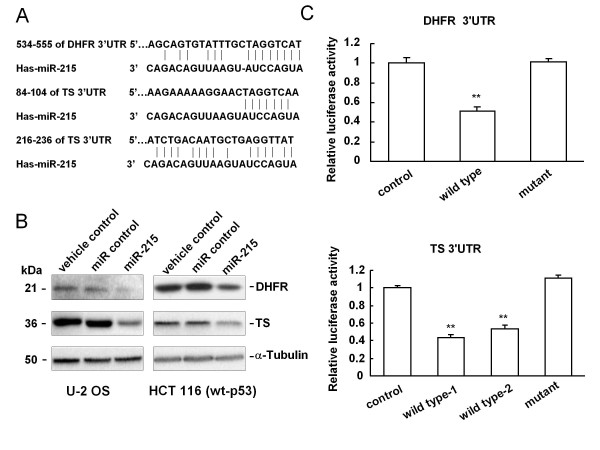
**DHFR and TS are the direct targets of miR-215**. (A) The 3'UTR of DHFR mRNA contains a putative binding site of miR-215. The 3'UTR of TS harbors two putative binding sites of miR-215. (B) The DHFR and TS protein levels were down-regulated in osteosarcoma U-2 OS and colon cancer HCT 116 (wt-p53) cells transfected with miR-215 (100 nM) by Western immunoblot analysis. Oligofectamine alone (vehicle control) and non-specific miRNA (miR control) were used as the negative controls. (C) The impacts of miR-215 on DHFR and TS expression by luciferase assays. HCT 116 (wt-p53) cells were cotransfected with 100 ng of pMIR-REPORT constructs (including wild-type DHFR or TS 3'UTRs and their corresponding mutant controls), 1 ng of Renilla luciferase plasmid phRL-SV40 and 100 nM of miR-215. Firefly luciferase activity for each condition was normalized by Renilla internal control. The value of relative luciferase activity for empty vector (control) was set as 1, the values for wild-type or mutant constructs were calculated as fold induction. ** *P *< 0.01, compared to the control, Student's *t *test (two-tailed). Each condition was repeated 3 times in triplicate and error bars represent standard deviations.

Given the significance of DHFR and TS as two of the major targets of anti-cancer chemotherapy, we used a bioinformatics approach to identify miRNAs that were predicted to bind to DHFR and TS mRNAs and therefore function in their regulation. Using TargetScan and PicTar in the miRNAs database (http://www.mirbase.org), we found that miR-215 was predicted to have a potential interaction site at the 3'UTR of DHFR mRNA and two sites at the 3'UTR of the TS mRNA (Figure 1A). Ectopic expression of miR-215 by transient transfection decreased the expression of both DHFR and TS proteins in both osteosarcoma U-2 OS and colon cancer HCT 116 (wt-p53) cells analyzed by Western immunoblot analysis (Figure 1B). However, only a slight reduction of DHFR or TS mRNAs expression was observed in HCT 116 (wt-p53) cells (Additional file 4A-B), and the results in U-2 OS cells were consistent with HCT 116 (wt-p53) cells (data not shown). This indicates that the suppression of DHFR or TS expression by miR-215 is, in a large part, reducing the protein translation. By contrast, the decreased expression of DHFR and TS by siRNAs (Additional file 5A-B) was clearly caused by mRNA degradation (Additional file 4A-B), and similar results were found in U-2 OS cells (data not shown). To provide direct evidence that miR-215 interacts with the 3'UTRs of DHFR or TS mRNAs, luciferase reporter constructs were prepared by inserting the DHFR or TS 3'UTRs containing the putative miR-215 binding sites into the downstream of the firefly luciferase reporter gene. Our results clearly demonstrated a significant decrease of luciferase activity compared to either mutant or empty vector controls upon transient over-expression of miR-215 (Figure 1C). Based on these results, we conclude that DHFR and TS are among some of the direct targets of miR-215.

Interestingly, a recent report describing a microarray expression analysis for miR-215 targets did not identify DHFR or TS [32], in a large part because the approach was dependent on mRNA target degradation. In fact, any analysis based on the examination of steady state total RNA levels would be likely to miss these important targets.

### miR-215 inhibits cell proliferation

**Figure 2:**
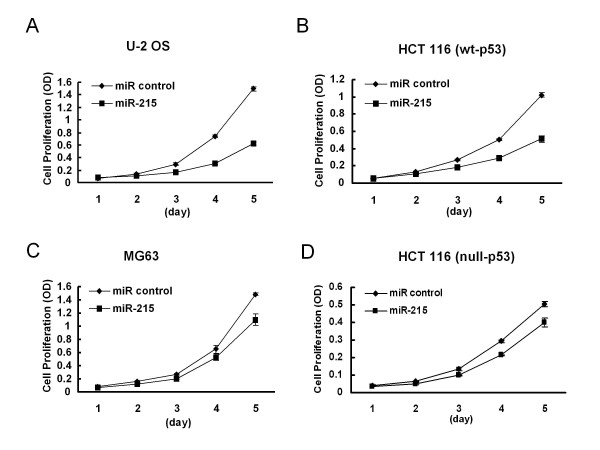
**miR-215 inhibits cell proliferation partly dependent on the p53 status in cancer cell lines**. Each cell type was transfected with 100 nM of miR control or miR-215 and cell numbers were determined by the WST-1 assays. miR control was used as the negative control. A remarkable inhibition of cell proliferation was observed in the p53 wild-type cell lines, U-2 OS and HCT 116 (wt-p53) (A and B), whereas much less effect was observed in the p53 mutant osteosarcoma MG63 cells or p53 knockout colon cancer HCT 116 (null-p53) cells (C and D). Numbers are indicated as mean ± SD.

Recent studies have clearly demonstrated that miRNAs play important roles in multiple biological processes, such as development, differentiation, cell proliferation, apoptosis, metabolism, and stress response, many of which are often perturbed in cancer [24,33]. Some miRNAs have been identified acting as either oncogenes or tumor suppressors, so to investigate the potential impact of miR-215 on cell growth, cell proliferation assays were performed in both U-2 OS and HCT 116 (wt-p53) cells. A significant inhibition of cell proliferation (over 40%) was observed in either U-2 OS or HCT 116 (wt-p53) cells compared with the non-specific miRNA control after 5 days (Figure 2A-B). The inhibitory effect was more profound in cells containing wild-type p53 than cells without functional p53 (Figure 2C-D), suggesting that this reduced cell proliferation may be caused by cell cycle arrest or cell senescence.

**Figure 3:**
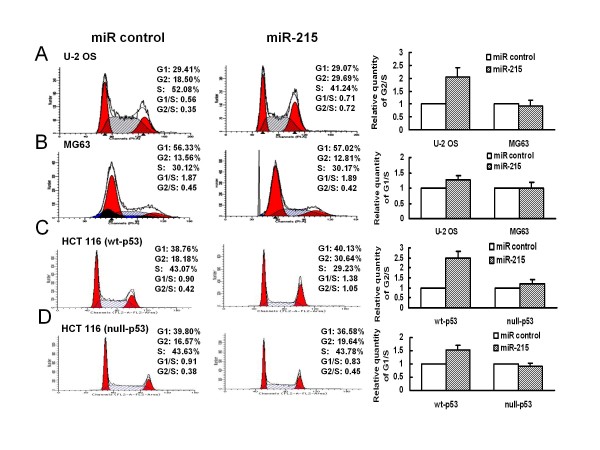
**miR-215 induces cell cycle p53-dependent G2-arrest**. The values of G1/S and G2/S ratio in the miR control were set as 1, the bar graphs showed the relative quantity of G1/S and G2/S ratio in the miR-215 transfected cells compared to the miR control as mean ± SD. This experiment was repeated two separate times, and similar results were obtained. The representative flow cytometry pattern was shown.

Specific miRNAs have been found to regulate cell cycle progression and apoptosis, which represents a new layer of complexity in the cell cycle regulation [34]. Here, we focused on the cell cycle changes induced by miR-215 through examining its impact on cell cycle control using flow cytometry. The proportion of cells in the G2 phase was higher in miR-215 transfected U-2 OS cells than that in non-specific miRNA control cells, whereas the proportion of cells in the S phase was much lower than that in the negative miRNA control cells, with the relative quantity of G2/S ratio >2-fold (Figure 3A). Similar results were observed in HCT 116 (wt-p53) colon cancer cells transfected with miR-215 (Figure 3C). Our results suggest that the reduced proliferation rate is due to the decreased S phase and increased G2 checkpoint control.

We also noticed that the effect of miR-215 on cell proliferation and cell cycle control were in part dependent on the status of p53, as the effect was much less in cell lines containing mutant or deleted p53 (Figure 2C-D and Figure 3B and 3D). This is consistent with recent reports that miR-215 contributes to cell cycle control and cell proliferation mediated by p53 [32,35]. It's known that p53 plays an important role in stem cell quiescence process [36]. We believe that we have also added a microRNA component to the p53-regulated network in stem cell quiescence.

### miR-215 increases the expression of cell cycle control genes p53 and p21 by down-regulation of DTL

**Figure 4:**
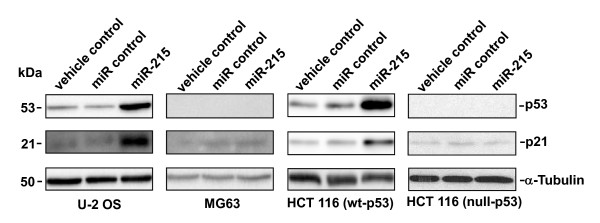
**miR-215 increases the expression of cell cycle control genes p53 and p21 in U-2 OS and HCT 116 (wt-p53) cells**. The expression of p53 and p21 was determined by Western immunoblot.

p53 and p21, a downstream target of the p53 growth control pathway, are reported to block cells at the G2 checkpoint mainly through inhibition of Cdc2 activity, the cyclin-dependent kinase that normally drives cells into mitosis and which is the ultimate target of pathways that mediate rapid arrest in G2 in response to DNA damage [37]. We found that miR-215 can induce G2-arrest in U-2 OS and HCT 116 (wt-p53) cells, so to further investigate the mechanism of cell proliferation inhibition by miR-215, we transfected miR-215 into U-2 OS and HCT 116 (wt-p53) cells and evaluated the levels of cell cycle control genes p53 and p21 by Western immunoblot analysis (Figure 4). The results showed that over-expression of miR-215 caused a significant increase of the p53 and p21 protein in both U-2 OS and HCT 116 (wt-p53) cells. Thus, our results indicate that miR-215 contributes to the inhibition of cell proliferation at least partially by the induction of G2-arrest in U-2 OS and HCT 116 (wt-p53) cells through over-expression of p53 and p21.

**Figure 5:**
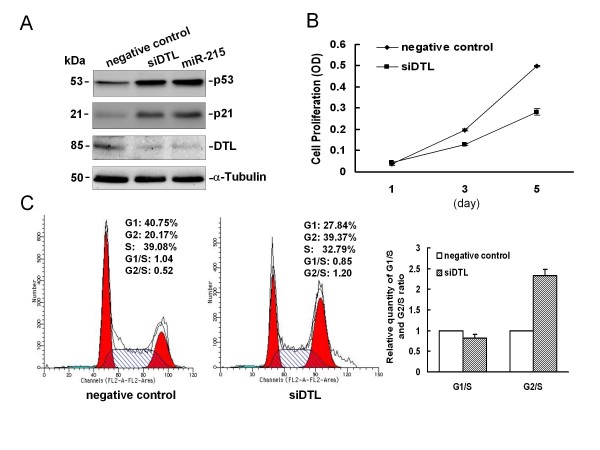
**miR-215 inhibits cell proliferation and triggers cell cycle G2-arrest by down-regulation of DTL and subsequent increased p53 and p21 in HCT 116 (wt-p53) cells**. (A) The DTL protein was down-regulated in colon cancer HCT 116 (wt-p53) cells transfected with miR-215 (100 nM) analyzed by Western immunoblot analysis, DTL specific siRNA (100 nM) was used as the positive control. (B) Suppression of DTL by DTL specific siRNA resulted in growth inhibition in HCT 116 (wt-p53) cells. Numbers are indicated as mean ± SD. (C) Down-regulation of DTL triggered cell cycle G2-arrest in HCT 116 (wt-p53) cells transfected with DTL specific siRNA. This experiment was repeated two separate times, and similar results were obtained. The representative flow cytometry pattern was shown.

We then asked what possibilities might contribute to the induction of p53 and p21 by miR-215. Georges et al. [32] reported that miR-215 induced cell cycle arrest by targeting a number of G1 and G2 checkpoint regulators. They confirmed that eighteen transcripts were direct downstream targets of miR-215, including denticleless protein homolog (DTL), a cell cycle G2/M checkpoint regulatory protein [28,38]. Moreover, DTL is thought to interact with both DDB1-CUL4 and MDM2-p53 ligase complexes [39,40]. Inactivation of DTL has been found to impair these complexes and stabilize p53 by preventing its ubiquitination, increasing the levels of p53 and its target p21 [28]. Here, we hypothesized that miR-215 might suppress DTL, the destabilizing factor of p53, to promote p53 stabilization and subsequently inhibit cell growth and cause cell cycle G2-arrest. First, we confirmed that miR-215 down-regulated DTL protein expression by Western immunoblot, and that knock-down of DTL by siRNA induced p53 and p21 expression to the same extent as miR-215 (Figure 5A). We next investigated the effects of DTL knock-down on cell proliferation and cell cycle in HCT 116 (wt-p53) cells, and found that cell proliferation was reduced significantly (about 43%) compared to negative control after 5 days (Figure 5B). Flow cytometry showed that the proportion of cells in the G2 phase was higher in HCT 116 (wt-p53) cells transfected with siRNA targeting DTL than that in negative control cells, while the proportion of cells in the S phase decreased, with the relative quantity of G2/S ratio >2-fold (Figure 5C). These results were similar to those observed in HCT 116 (wt-p53) cells transfected with miR-215 (Figure 2B and 3C). Taken together, our results indicate that miR-215 inhibits cell proliferation by the induction of G2-arrest through down-regulation of G2 checkpoint regulator DTL and up-regulation of p53 and p21.

### Reduced chemosensitivity to MTX or TDX by miR-215 is caused by the reduction of DTL expression

**Figure 6:**
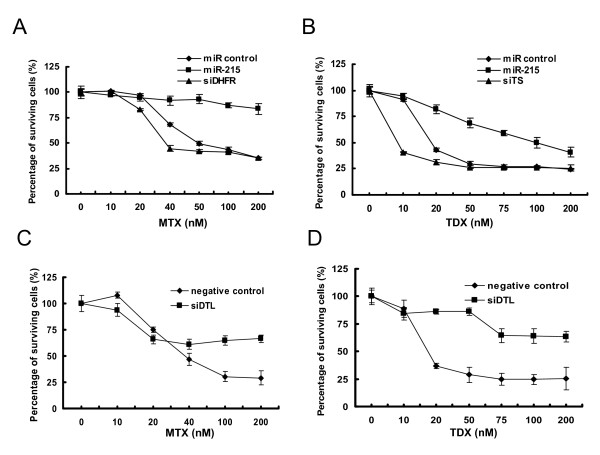
**miR-215 reduces the chemosensitivity to DHFR inhibitor MTX (A) or TS inhibitor TDX (B) in the HCT 116 (wt-p53) cells via targeting DTL (C and D)**. HCT 116 (wt-p53) cells transfected with miR-215 or siRNAs specific for DHFR, TS or DTL were incubated with MTX or TDX ranged from 10-200 nM for 72 h, and WST-1 assays were performed. siRNAs specific against DHFR, TS or DTL were positive controls.

To evaluate the impact of miR-215 on chemosensitivity, we used MTX or TDX to treat HCT 116 (wt-p53) cells transfected with either miR-215, non-specific miRNA, or siRNAs against DHFR or TS. Examination revealed that cells transfected with siRNAs specific for DHFR or TS showed a considerably heightened sensitivity to both MTX and TDX (Figure 6A-B). This is consistent with previous reports that tumors with lower expression of DHFR or TS are more sensitive to antifolate treatment [1,3,30,41]. However, the cells transfected with miR-215 were considerably less sensitive to both chemotherapeutic agents than were control cells (Figure 6A-B). Similar results were obtained with U-2 OS cells (data not shown). Van Triest et al. [42] and Peters et al. [43] found there is no significant relationship between the TS levels and TDX sensitivity and suggested that the sensitivity to the TS-directed antifolate is unlikely to be determined by one single determinant. Since miR-215 potentially regulates hundreds of mRNA transcripts, its global impact on genes and pathways has the potential to be more important for the resistance mechanism. Based on the results of cell proliferation and cell cycle between miR-215 and siRNAs against DHFR or TS, we reasoned that the opposite impact of miR-215 on chemosensitivity vs. siRNAs specific to DHFR or TS may be largely due to the reduced cell proliferation rate (Figure 2) through decreased S phase and increased G2-arrest (Figure 3). MTX and TDX are considered to be cell cycle-specific agents and mainly affect cells in the S phase [43-47]. In general, slowly proliferating cells are far more resistant to chemotherapeutic drug treatment, particularly slowly proliferating tumor stem cells [29]. Since siRNAs specific for DHFR and TS reduced the levels of their targets without affecting the rate of cell proliferation, they greatly enhanced the toxicity of DHFR and TS inhibitors (Additional file 5C-D). To identify the possible target(s) of miR-215 responsible for the reduced cell proliferation and cell cycle G2-arrest, we investigated several miR-215-mediated targets with a role in cell cycle control (data not shown). As mentioned above, we discovered that one of them, DTL, was directly responsible for G2-arrest and the induction of p53 and p21 (Figure 5). We then investigated the impact of DTL expression on MTX and TDX chemosensitivity in HCT 116 (wt-p53) cells by performing siRNA-mediated knock-down of DTL. When cells were transfected with siRNA targeting DTL, they exhibited a significant increase in chemoresistance to both MTX and TDX (Figure 6C-D) similar to miR-215 (Figure 6A-B). Taken together, our results suggest that miR-215 may affect chemoresistance to MTX and TDX by suppressing DTL expression, thereby increasing G2-arrest and reducing the proportion of time spent in S phase, during which MTX and TDX are most effective.

We reasoned that if our conclusion was correct, and that the mechanism of miR-215-mediated resistance lies in its ability to trigger G2-arrest with slow cell proliferation, then altering miR-215 levels should have no effect on the toxicity of agents whose actions are cell cycle-independent. To test this, we repeated the previous drug treatment experiments in HCT 116 (wt-p53) cells, but replacing MTX and TDX with the DNA-targeting agents-cisplatin and doxorubicin. As shown in Additional file 6A-B, no significant difference in chemosensitivity was observed between miR-215 transfected cells and negative control cells. Similar results were also found in cells transfected with siRNA against DTL (Additional file 6C-D). These results further support our conclusion that miR-215-mediated MTX and TDX resistance is due to its effects on the cell cycle and suggest that the resistance mechanism mediated by miR-215 is specific to cell cycle-dependent drugs.

### Impact of endogenous miR-215 on cell proliferation, cell cycle and chemosensitivity

We have so far accessed the functional significance of miR-215 using a knock-in approach. This, to a certain extent, mimics a cellular stress response in which miR-215 is induced. The results showed that exogenous miR-215 reduced cell proliferation with increased cell cycle control and chemoresistance. To further elucidate the impact of endogenous miR-215 on cell proliferation, cell cycle and chemosensitivity, we performed a series of knock-down experiments using locked nucleic acid (LNA) oligonucleotides (a scramble-miR LNA negative control, and a LNA antisense miR-215) to test the biological significance of endogenous miR-215 in HCT 116 (wt-p53) cells. Antagonizing the endogenous miR-215 enhanced the cell proliferation rate by 23% compared to the LNA negative control (Additional file 7A), and increased the sensitivity to MTX treatment (Additional file 7B). The expression of TS and DHFR were increased by knocking down miR-215 using Western immunoblot analysis (Additional file 7C). These observations further demonstrate the important effects of endogenous miR-215 on cell proliferation and chemosensitivity.

To test whether we can reverse the cell cycle impact caused by exogenous miR-215, we antagonized miR-215 by transfecting cells with 100 nM LNA antisense miRNAs. We observed that the percentage of cells in the G2 phase decreased from 37% to 24%, and percentage of cells in the S phase increased from 19% to 34% (Additional file 8A, top panel) in the HCT 116 (wt-p53) cell line, while HCT 116 (null-p53) cells showed no change in the G2 and S phases (Additional file 8A, bottom panel). These results further support the notion that miR-215 is important in regulating the cell cycle in a manner of depending on p53 status. Strikingly, antagonizing miR-215 also attenuated the induction of p53 and p21 (Additional file 8B). These results are highly consistent with the data obtained from exogenous miR-215 over-expression experiments.

### Elevated expression of miR-215 in human colon cancer stem cells may contribute to chemoresistance

Cancer stem cells (CSC), as their name implies, are cancer cells that possess the characteristics associated with normal stem cells, in particular the ability to give rise to all cell types found in a particular cancer sample. In contrast with other more differentiated cancers, however, CSCs exhibit a low rate of division and proliferation that allows them to resist chemotherapies and radiation [29], both of which preferentially affect highly proliferative cells, making CSCs a major reason for the failure of chemotherapy. With this in mind, we analyzed the miR-215 expression levels from isolated CD133+HI/CD44+HI colon cancer stem cells from cultured HCT 116 (wt-p53) cells using real time qRT-PCR analysis (Additional file 9B). We used CD133 and CD44 as two selection markers to isolate colon cancer stem cells from HCT 116 (wt-p53) cells (Additional file 9A) because CD133 and CD44 have been shown to be two of the important markers for the isolation of colon cancer stem cells [48-51]. The details of characterization of CD133+HI/CD44+HI colon cancer stem cells have been previously reported [51]. Expression of miR-215 in the CD133+HI/CD44+HI colon cancer stem cells was nearly 3-fold higher than that in the control bulk CD133+/CD44+ colon cancer cells (Additional file 9B). These results suggest that colon cancer stem cells may utilize miR-215 to slow cell proliferation and avoid damage caused by chemotherapy until receiving a proliferation and differentiation signal, further verifying the impact of miR-215 on cell proliferation and chemotherapy resistance. To further confirm that DHFR and TS are the targets of miR-215, the expression of both DHFR and TS were quantified in CD133+HI/CD44+HI colon cancer stem cells and the control bulk CD133+/CD44+ colon cancer cells. We found DHFR and TS protein levels were remarkably down-regulated in the CD133+HI/CD44+HI colon cancer stem cells based on the relative higher miR-215 expression level (Additional file 9C). This result, in turn, suggests that miR-215 is more important than the levels of DHFR or TS in the chemoresistance.

### Decreased expression of miR-215 in human colorectal cancer specimens

Previous studies from our laboratory have shown that certain miRNAs were associated with the development and prognosis in colorectal cancer [52]. To provide potential relevance of miR-215 in colorectal cancer, we profiled the expression of miR-215 in the same set of clinical samples (24 colorectal tumor specimens vs. adjacent normal colorectal tissues) using real time qRT-PCR analysis. The expression of miR-215 was significantly decreased (*P *< 0.01) in colorectal tumor specimens compared to adjacent normal tissues (Additional file 10). These results indicate that the fast proliferating phenotype in the majority of differentiated colorectal tumor cells are associated with the reduction of miR-215 expression. This further supports our hypothesis that the small fraction of tumor stem cells with a slow proliferation rate is mediated, at least in part, by miR-215. Based on these findings, it is clear that the roles miR-215 plays in regulating cellular behavior are too complex for it to be simply defined as a tumor suppressor based on its ability to slow cell proliferation and cause cell cycle arrest under certain conditions, and it has to be defined in the right cellular context.

## Conclusions

Taken together, our results clearly indicate that miR-215 over-expression results in the resistance to DHFR inhibitor MTX or TS inhibitor TDX treatment. This is achieved largely by the reduced proliferation rate and cell cycle arrest mediated by miR-215 through down-regulation of DTL, despite the fact that miR-215 also down-regulates the expression of both DHFR and TS. The elevated expression of miR-215 in colon cancer stem cells with slow proliferation rate and resistance to chemotherapy further supports the roles of miR-215 in cell proliferation and chemotherapy resistance. This study provides a novel mechanism of chemoresistance mediated by miR-215, suggesting that it may have a unique potential as a novel biomarker candidate.

## Competing interests

The authors declare that they have no competing interests.

## Authors' contributions

JJ designed research; BS and YW performed research; BS, YW, MAT, and GB analyzed data; A. and MK provided the clinical colorectal cancer samples; BS, MAT, and JJ wrote the paper. All the authors read and approved the final manuscript.

## Supplementary Material

Additional file 1 **Supplementary Table 1**. Characteristics of the 24 colorectal cancer patients. Click here for file

Additional file 2 **Methods**. Document detailing the methods used. Click here for file

Additional file 3 **Supplementary Table 2**. Sequences of synthesized oligonucleotides for the miR-215 binding site(s) of DHFR and TS. Click here for file

Additional file 4 **Real time qRT-PCR analysis of DHFR mRNA (A) or TS mRNA levels (B) in HCT-116 (wt-p53) cells transfected with miR-215 or siRNAs specific for DHFR or TS**. Oligofectamine alone (vehicle control) and non-specific siRNA (negative control) were negative controls, the value of DHFR mRNA or TS mRNA in the negative control was set at 1, the relative amount in siRNAs against DHFR and TS or miR-215 transfected cells was indicated as fold induction. *P < 0.05, compared to the negative control, Student's *t *test (two-tailed). Each condition was repeated 3 times and error bars represent standard deviations. Click here for file

Additional file 5 **Cells transfected with DHFR or TS gene specific siRNAs maintain similar proliferation rate compared to the negative control**. (A and B) Western immunoblot analysis of protein expression levels of DHFR and TS by siRNAs against DHFR or TS. (C and D) The impacts of siRNAs against DHFR or TS on the cell proliferation. Non-specific siRNA (negative control) was used as the negative control. Click here for file

Additional file 6 **miR-215 has no effect on the cytotoxicity of cisplatin and doxorubicin**. HCT 116 (wt-p53) cells transfected with miR-215 mimics, non-specific miRNA, or non-targeting siRNA and siRNA against DTL were incubated with cispaltin (0.625-10 μM) or doxorubicin (25-500 nM) for 72 h and cell viability was measured by WST-1 at 450 and 630 nm respectively. Click here for file

Additional file 7 **Knock-down of endogenous miR-215 enhances the cell proliferation and chemosensitivity to MTX**. (A) HCT 116 (wt-p53) cells were transfected with 100 nM of scramble-miR locked nucleic acid (LNA-control) or LNA anti-miR215 oligonucleotide (LNA-miR215) by Lipofectamine 2000, cell proliferation analysis was performed as described in Additional file 2. (B) HCT 116 (wt-p53) cells were transfected with LNA-control or LNA-miR215 and treated with MTX for 72 h, viable cells were accessed by WST-1 assays. (C) Proteins were extracted at 48 h after transfection with LNA-miR215 and subjected to Western immunoblot analysis to detect DHFR and TS, LNA-control was used as the negative control. Click here for file

Additional file 8 **Antagonizing miR-215 by LNA anti-miR reverses the impact of miR-215 on the cell cycle**. (A) Knock-down miR-215 decreased G2 phase and increased S phase of the cell cycle in HCT 116 (wt-p53) cells, no such effects were found in HCT 116 (null-p53) cells. HCT 116 (wt-p53) cells and HCT 116 (null-p53) cells were transfected with 100 nM miR-215 for 24 h. Cell cycle analysis was performed after transfected with 100 nM LNA-miR215 for 48 h. (B) In parallel, LNA-miR215 prevented the induction of p53 and p21 expression in HCT 116 (wt-p53) cells analyzed by Western immunoblot analysis. LNA-control was the negative control. This experiment was repeated two separate times, and similar results were obtained. The representative flow cytometry pattern was shown. Click here for file

Additional file 9 **The expression of miR-215 in human colon cancer stem cells is elevated**. (A) FACS analysis was performed to sort colon cancer stem cells using CD133 and CD44 as the markers. CD133+HI/CD44+HI cells were considered as the colon cancer stem cells. CD133+/CD44+ and CD133NEG/CD44NEG were considered as the colon cancer cells. (B) Expression of miR-215 in human colon cancer stem cells was analyzed by real-time qRT-PCR. The value of miR-215 in the CD133+/CD44+ colon cancer cells was set at 1, the relative amount in CD133+HI/CD44+HI colon cancer stem cells and CD133NEG/CD44NEG colon cancer cells was showed as the fold induction. (C) The expression of DHFR and TS proteins was decreased in CD133+HI/CD44+HI colon cancer stem cells compared to control cell population analyzed by Western immunoblot analysis. Click here for file

Additional file 10 **miR-215 expression is decreased in colorectal cancer compared to normal colorectal specimens by real time qRT-PCR analysis**. Expression level of miR-215 was normalized by the internal control RNU6B in each sample. *P *= 0.0002, two-tailed paired Wilcoxon test. Click here for file
