# Supplementary material for: Molecular mechanism of chemoresistance by miR-215 in osteosarcoma and colon cancer cells
Source: Mol Cancer. 2010 Apr 30;9:96. doi: 10.1186/1476-4598-9-96 (PMC2881118; doi:10.1186/1476-4598-9-96)
Supplement: Additional file 1 — Supplementary Table 1. Characteristics of the 24 colorectal cancer patients. [file 1476-4598-9-96-S1.DOC]

**Table 1. Characteristics of the 24 colorectal caner patients.**

| **Characteristics** | | **Frequency** | **Percentage (%)** |
| --- | --- | --- | --- |
| **Age (Years)** | |  |  |
|  | Mean (range) | 62 (30-93) |  |
| **Gender** | |  |  |
|  | Male | 14 | 58.3 |
|  | Female | 10 | 41.7 |
| **Anatomic site** | |  |  |
|  | Ascending colon | 3 | 12.5 |
|  | Transverse colon | 2 | 8.3 |
|  | Descending colon | 4 | 16.7 |
|  | Sigmoid colon | 3 | 12.5 |
|  | Rectum | 12 | 50.0 |
| **Histology** | |  |  |
|  | Adenocarcinoma | 24 | 100 |
| **UICC stage** | |  |  |
|  | I | 4 | 16.7 |
|  | II | 4 | 16.7 |
|  | III | 8 | 33.3 |
|  | IV | 8 | 33.3 |
