# Supplementary material for: Molecular mechanism of chemoresistance by miR-215 in osteosarcoma and colon cancer cells
Source: Mol Cancer. 2010 Apr 30;9:96. doi: 10.1186/1476-4598-9-96 (PMC2881118; doi:10.1186/1476-4598-9-96)
Supplement: Additional file 2 — Methods. Document detailing the methods used. [file 1476-4598-9-96-S2.DOC]

**Additional file 2-Methods**

**RNA isolation**

Total RNAs, including miRNAs, were isolated from the cell lines, CD133+HI/CD44+HI colon cancer stem cells, CD133+/CD44+ and CD133NEG/CD44NEG colon cancer cells using TRIzol reagent (Invitrogen) according to the manufacturer’s instructions. Same method was also used to isolate total RNAs from colorectal cancer specimens and adjacent normal tissues.

**Real time qRT-PCR analysis of miR-215**

cDNA synthesis was carried out with the High Capacity cDNA synthesis kit (Applied Biosystems). Real-time quantitative reverse transcription-PCR (qRT-PCR) analysis was performed on an Applied Biosystems 7500 Real-Time PCR System. The miRNA sequence-specific RT-PCR primers for miR-215 and endogenous control RNU6B were purchased from Ambion. The gene expression Δ*C*_T_ values of miRNAs from each sample were calculated by normalizing with internal control RNU6B and relative quantitation values were plotted.

**Real time qRT-PCR analysis of mRNA expression**

cDNA was synthesized with the High Capacity cDNA synthesis kit (Applied Biosystems) using random primers. The PCR primers and probes for DHFR, TS and the internal control gene GAPDH were purchased from Applied Biosystems. qRT-PCR was performed on an ABI 7500HT instrument (Applied Biosystems) by Taqman Gene Expression Assay under the following conditions: 50°C, 2 min of reverse transcription; 95°C, 10 min; 95°C, 15 s; 60°C, 1 min for up to 40 cycles (n =3).

**Cell proliferation analysis**

Cells were replated in 96-well plates in triplicate at 1×10^3^ cells/well after transfection with miRNA precusors, siRNAs against DHFR, TS or DTL, or locked nucleic acid (LNA) antisense miRNAs. Cells were cultured for extra 24, 48, 72, 96 h. The absorbance at 450 and 630 nm was measured after incubation with 10 μl of WST-1 (Roche Applied Science) for 2 h.

**Cell cycle analysis**

U-2 OS, MG63, HCT 116 (wt-p53) and HCT 116 (null-p53) cells were transfected with miRNA precursors or siRNAs described as above. At 36 h after transfection, cells were harvested and resuspended at 0.5-1×10^5^ cells/ml in modified Krishan buffer containing 0.1% sodium citrate and 0.3% NP-40 [1, 2] and kept at 4°C. Before being analyzed by flow cytometry, cells were treated with 0.02 mg/ml RNase H and stained with 0.05 mg/ml propidium iodide (Sigma). HCT 116 (wt-p53) and HCT 116 (null-p53) cells were collected at 48 h after transfection with LNA antisense miRNAs for flow cytometry analysis described as above.

**Western immunoblot analysis and antibodies**

After transfection with miRNA precursors, siRNAs, or LNA antisense miRNAs, the cells were scraped and lysed in RIPA buffer (Sigma). CD133+HI/CD44+HI and CD133+/CD44+ cells were lysed in RIPA buffer and the proteins were extracted. The primary antibodies included mouse anti-DHFR mAb (1:250, BD Bioscience), mouse anti-TS mAb (1:400, Millipore), rabbit anti-DTL polyclonal Ab (1:1000, BETHYL), mouse anti-p53 mAb (1:1000, DO-1), mouse anti-p21 mAb (1:1000, F-5), and mouse anti-α-tubulin mAb (1:1000, TU-02) from Santa Cruz Biotechnology.

**Plasmid construction, transfection and luciferase assays**

Twenty-four hours before transfection, U-2 OS and HCT 116 (wt-p53) cells were plated in the 96-well plates at 1.5×10^4^ cells/well in triplicate. pMIR-REPORT constructs (100 ng) together with 1 ng of Renilla luciferase plasmid phRL-SV40 (Promega) and 100 nM of miR-215 were transfected by Lipofectamine 2000 (Invitrogen). Thirty hours after transfection, cells were lysated and luciferase activity was measured by the dual-luciferase reporter assay system (Promega). Firefly luciferase activity for each condition was normalized by dividing to Renilla internal control and then compared to empty vector pMIR-REPORT.

**Cisplatin and doxorubicin chemosensitivity**

HCT 116 (wt-p53) cells were replated in 96-well plates at 2×10^3^ cells/well in triplicate after transfected with miR-215 mimics, non-specific miRNA, or non-targeting siRNA (Dharmacon) and siRNA against DTL in 100 μl of medium. Twenty-four hours later, cispaltin (0.625-10 µM) or doxorubicin (25-500 nM) in 100 μl medium was added, and incubated for 72 h. WST-1 was added to each well (10 μl). After 2 h incubation, absorbance was measured at 450 and 630 nm respectively.

References:

1. Dressler LG, Seamer LC, Owens MA, Clark GM, McGuire WL: **DNA flow cytometry and prognostic factors in 1331 frozen breast cancer specimens.** *Cancer* 1988, **61:**420-427.
2. Krishan A: **Rapid flow cytofluorometric analysis of mammalian cell cycle by propidium iodide staining.** *J Cell Biol* 1975, **66:**188-193.
