# Supplementary material for: Molecular mechanism of chemoresistance by miR-215 in osteosarcoma and colon cancer cells
Source: Mol Cancer. 2010 Apr 30;9:96. doi: 10.1186/1476-4598-9-96 (PMC2881118; doi:10.1186/1476-4598-9-96)
Supplement: Additional file 3 — Supplementary Table 2. Sequences of synthesized oligonucleotides for the miR-215 binding site(s) of DHFR and TS. [file 1476-4598-9-96-S3.DOC]

**Table 2.** **Sequences of synthesized oligonucleotides for miR-215 binding site(s)**

**of DHFR and TS.**

| 3’UTR | Location | Sequences of oligonucleotides |
| --- | --- | --- |
| DHFR mRNA | 519-578bp | Forward-wild type:  5’CTAGTAATTTCAGTGAAAGCAGTGTATTTGCTAGGTCATACCAGAAATCATCAATTGAGGTACGGA3’  Reverse-wild type:  5’AGCTTCCGTACCTCAATTGATGATTTCTGGTATGACCTAGCAAATACACTGCTTTCACTGAAATTA3’  Forward-mutant:  5’CTAGTAATTTCAGTGAAAGCAGTGT*GC*TTGC*G*A*TA*T*G*ATACCAGAAATCATCAATTGAGGTACGGA3’  Reverse-mutant:  5’AGCTTCCGTACCTCAATTGATGATTTCTGGTATCATATCGCAAGCACACTGCTTTCACTGAAATTA3’ |
| TS mRNA | 198-247bp | Forward-wild type-1: 5’CTAGTAGTTAACTCCCTGAGGGTATCTGACAATGCTGAGGTTATGAACAAAGTGA3’  Reverse-wild type-1: 5’AGCTTCACTTTGTTCATAACCTCAGCATTGTCAGATACCCTCAGGGAGTTAACTA3’  Forward-mutant: 5’CTAGTAGTTAACTCCCTGAGGGTAT*A*T*C*AC*G*ATG*T*TGA*TA*T*C*A*C*GAACAAAGTGA3’  Reverse-mutant: 5’AGCTTCACTTTGTTCGTGATATCAACATCGTGATATACCCTCAGGGAGTTAACTA3’ |
| TS mRNA | 62-115bp | Forward-wild type-2: 5’CTAGTAGTTCTTTTTGCTCTAAAAGAAAAAGGAACTAGGTCAAAAATCTGTCCGA3’  Reverse-wild type-2: 5’AGCTTCGGACAGATTTTTGACCTAGTTCCTTTTTCTTTTAGAGCAAAAAGAACTA3’ |

Underlined base pairs are the predicted binding sites; italic base pairs are mutant.
