# Supplementary material for: Molecular mechanism of chemoresistance by miR-215 in osteosarcoma and colon cancer cells
Source: Mol Cancer. 2010 Apr 30;9:96. doi: 10.1186/1476-4598-9-96 (PMC2881118; doi:10.1186/1476-4598-9-96)
Supplement: Additional file 4 — Real time qRT-PCR analysis of DHFR mRNA (A) or TS mRNA levels (B) in HCT-116 (wt-p53) cells transfected with miR-215 or siRNAs specific for DHFR or TS. Oligofectamine alone (vehicle control) and non-specific siRNA (negative control) were negative controls, the value of DHFR mRNA or TS mRNA in the negative control was set at 1, the relative amount in siRNAs against DHFR and TS or miR-215 transfected cells was indicated as fold induction. *P < 0.05, compared to the negative control, Student's t test (two-tailed). Each condition was repeated 3 times and error bars represent standard deviations. [file 1476-4598-9-96-S4.PPT]

## Slide 1
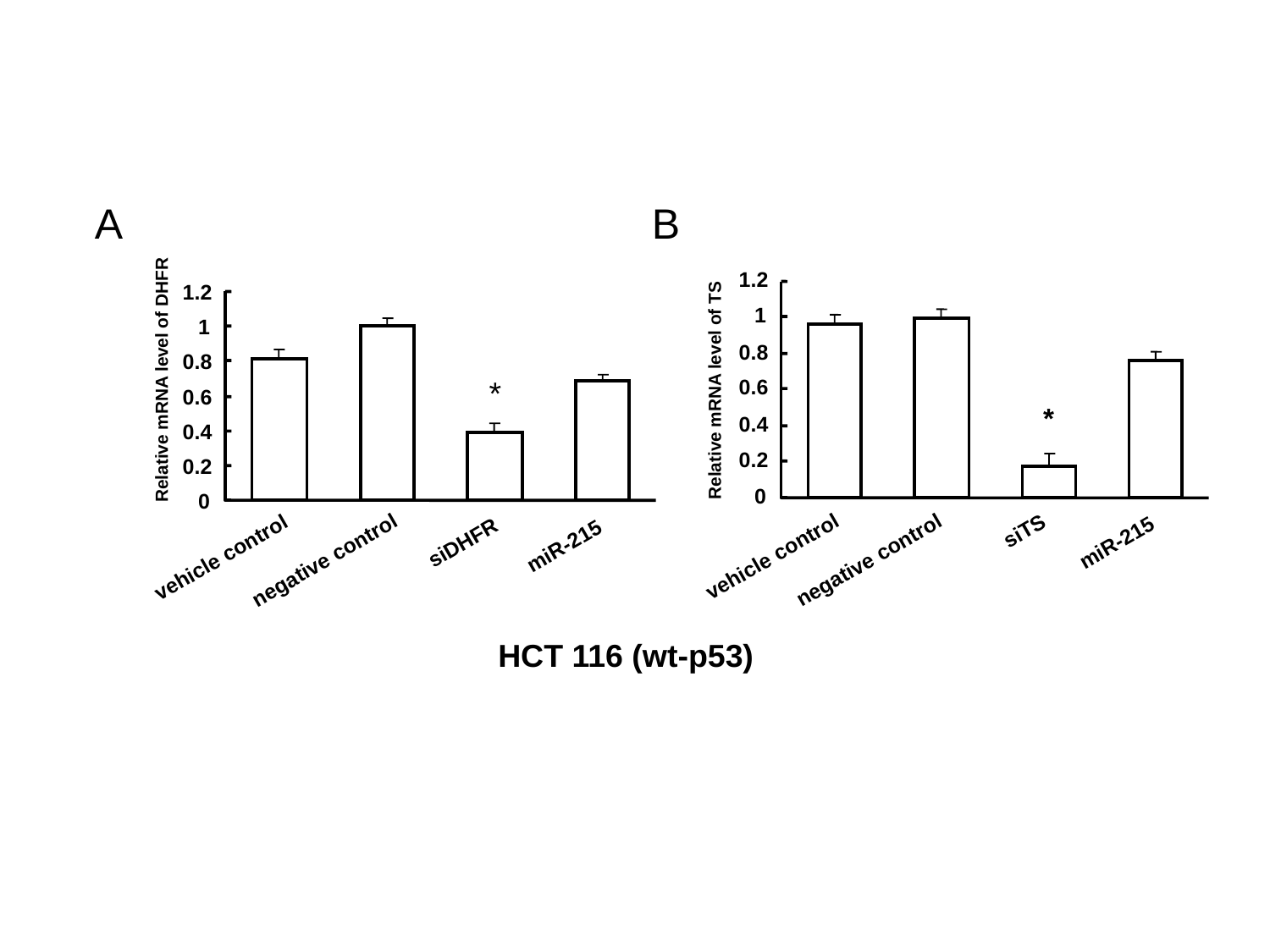

A B
1.2
1.2
1
1
0.8
0.8
Relative mRNA level of DHFR
Relative mRNA level of TS
*
0.6
0.6
*
0.4
0.4
0.2
0.2
0
0
siTS
miR-215
siDHFR
miR-215
vehicle control
vehicle control
negative control
negative control
HCT 116 (wt-p53)
