# Supplementary material for: Molecular mechanism of chemoresistance by miR-215 in osteosarcoma and colon cancer cells
Source: Mol Cancer. 2010 Apr 30;9:96. doi: 10.1186/1476-4598-9-96 (PMC2881118; doi:10.1186/1476-4598-9-96)
Supplement: Additional file 5 — Cells transfected with DHFR or TS gene specific siRNAs maintain similar proliferation rate compared to the negative control. (A and B) Western immunoblot analysis of protein expression levels of DHFR and TS by siRNAs against DHFR or TS. (C and D) The impacts of siRNAs against DHFR or TS on the cell proliferation. Non-specific siRNA (negative control) was used as the negative control. [file 1476-4598-9-96-S5.PPT]

## Slide 1
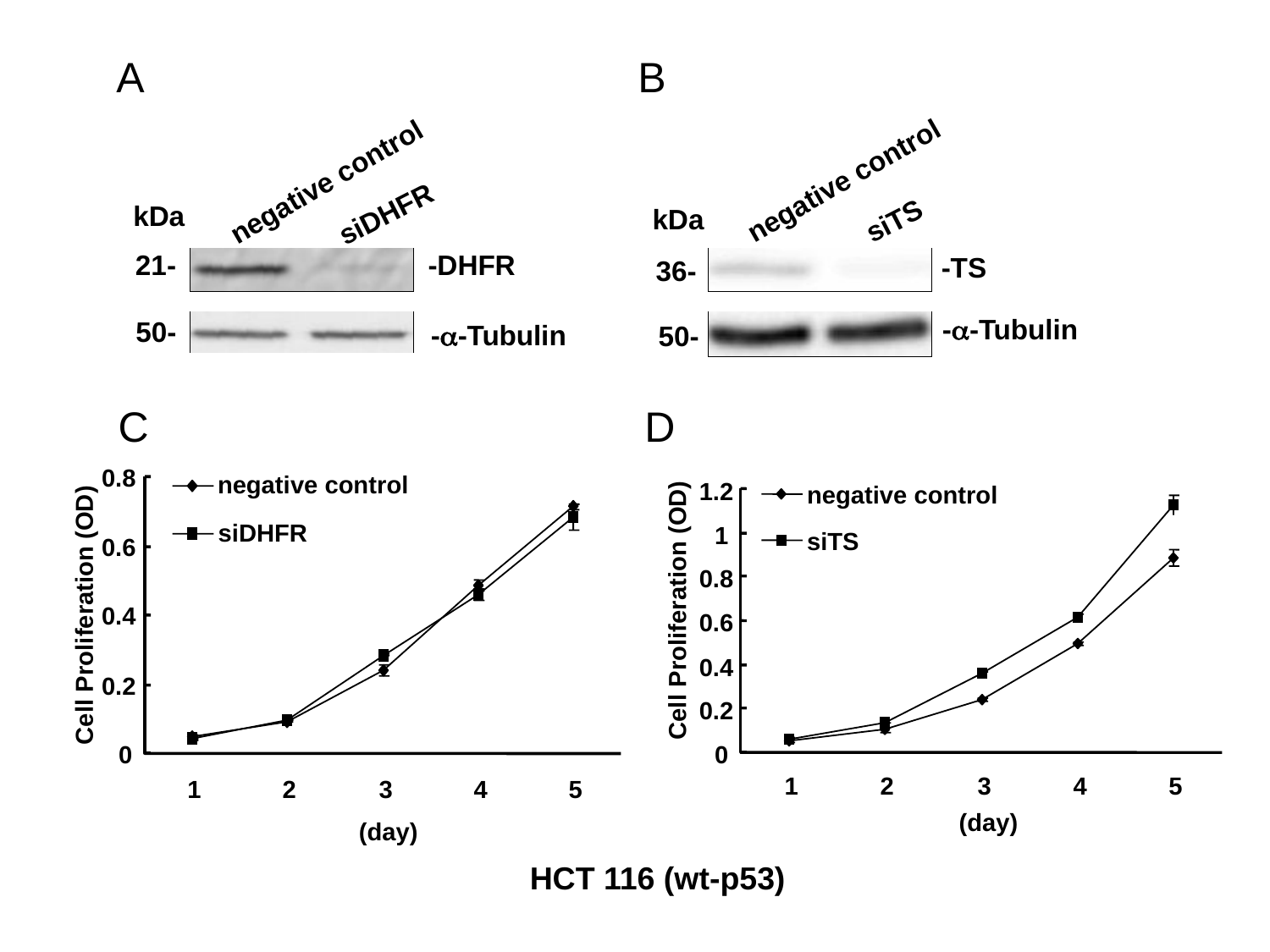

A B
negative control
siDHFR
kDa
21-
-DHFR
50-
-α-Tubulin
negative control
kDa
siTS
-TS
36-
-α-Tubulin
50-
C D
0.8
negative control
siDHFR
0.6
0.4
Cell Proliferation (OD)
0.2
0
1
2
3
4
5
(day)
1.2
negative control
1
siTS
0.8
Cell Proliferation (OD)
0.6
0.4
0.2
0
1
2
3
4
5
(day)
HCT 116 (wt-p53)
