# Supplementary figures and images for: Molecular mechanism of chemoresistance by miR-215 in osteosarcoma and colon cancer cells
Source: Mol Cancer. 2010 Apr 30;9:96. doi: 10.1186/1476-4598-9-96 (PMC2881118; doi:10.1186/1476-4598-9-96)

## Slide 1
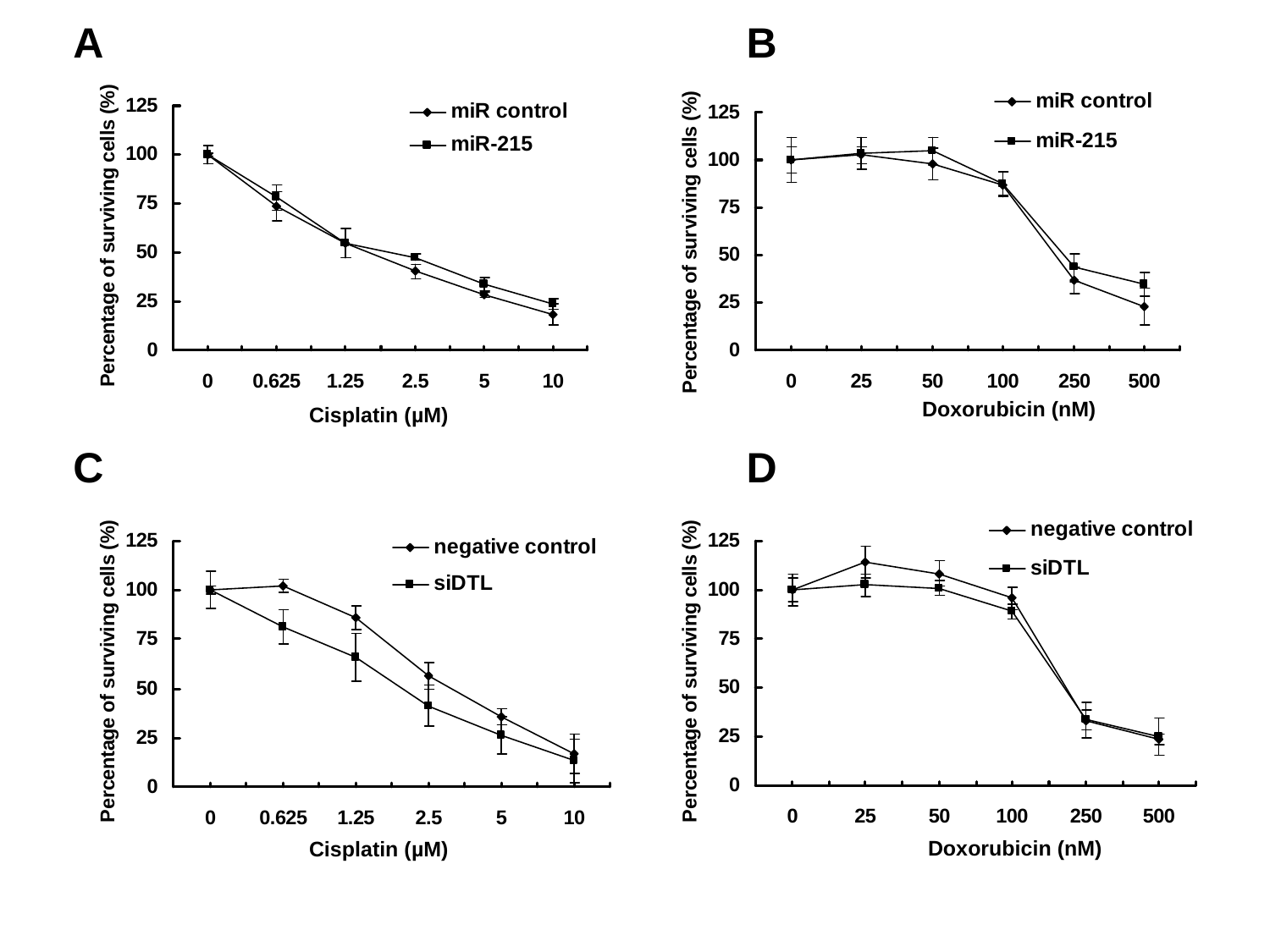

A				 B
Doxorubicin (nM)
Cisplatin (µM)
C				 D
Doxorubicin (nM)
Cisplatin (µM)

Supplement: Additional file 6 — miR-215 has no effect on the cytotoxicity of cisplatin and doxorubicin. HCT 116 (wt-p53) cells transfected with miR-215 mimics, non-specific miRNA, or non-targeting siRNA and siRNA against DTL were incubated with cispaltin (0.625-10 μM) or doxorubicin (25-500 nM) for 72 h and cell viability was measured by WST-1 at 450 and 630 nm respectively. [file 1476-4598-9-96-S6.PPT]

## Slide 1
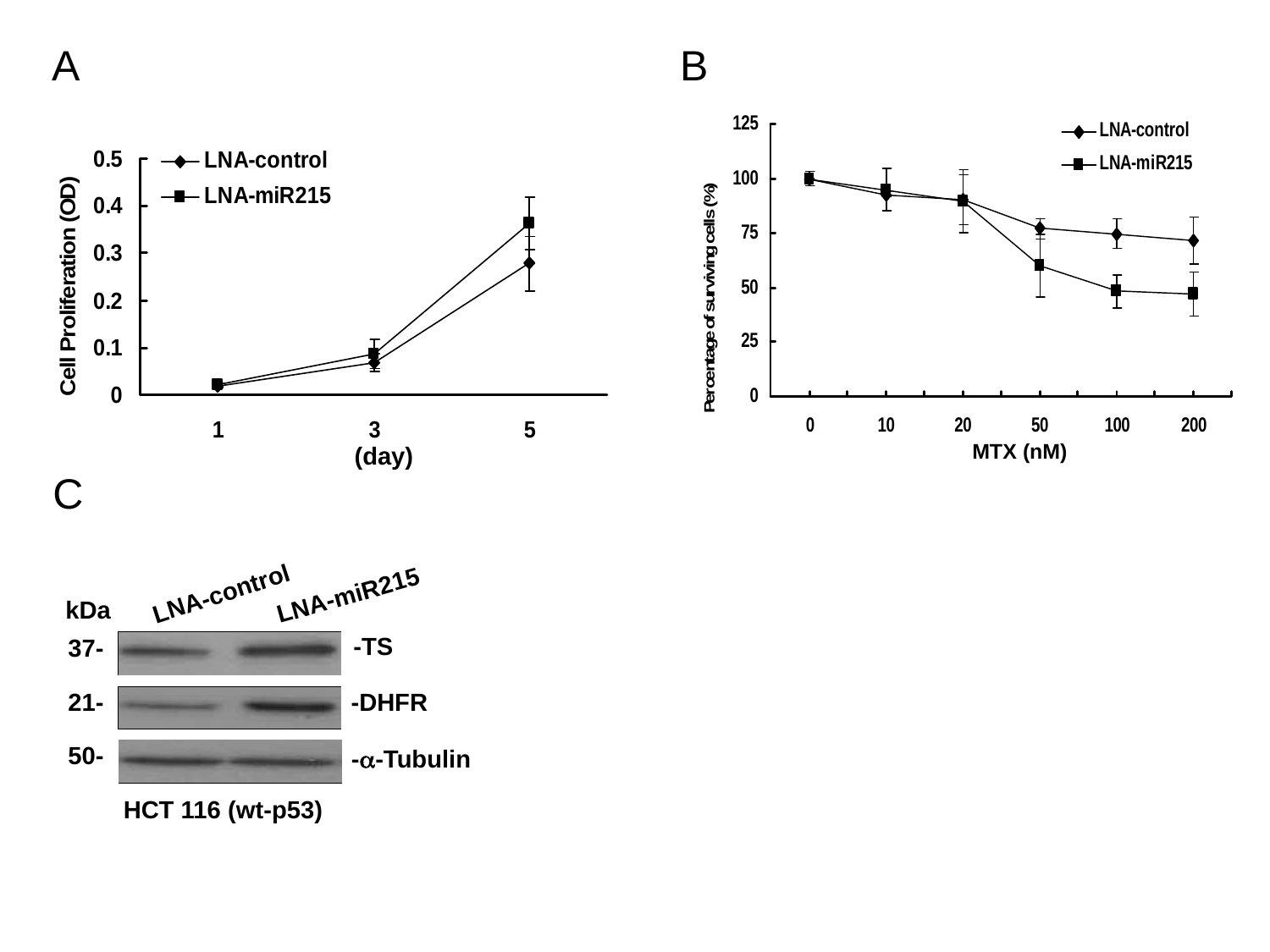

A B
MTX (nM)
(day)
C
LNA-control
LNA-miR215
kDa
-TS
-DHFR
-α-Tubulin
37-
21-
50-
HCT 116 (wt-p53)

Supplement: Additional file 7 — Knock-down of endogenous miR-215 enhances the cell proliferation and chemosensitivity to MTX. (A) HCT 116 (wt-p53) cells were transfected with 100 nM of scramble-miR locked nucleic acid (LNA-control) or LNA anti-miR215 oligonucleotide (LNA-miR215) by Lipofectamine 2000, cell proliferation analysis was performed as described in Additional file 2. (B) HCT 116 (wt-p53) cells were transfected with LNA-control or LNA-miR215 and treated with MTX for 72 h, viable cells were accessed by WST-1 assays. (C) Proteins were extracted at 48 h after transfection with LNA-miR215 and subjected to Western immunoblot analysis to detect DHFR and TS, LNA-control was used as the negative control. [file 1476-4598-9-96-S7.PPT]

## Slide 1
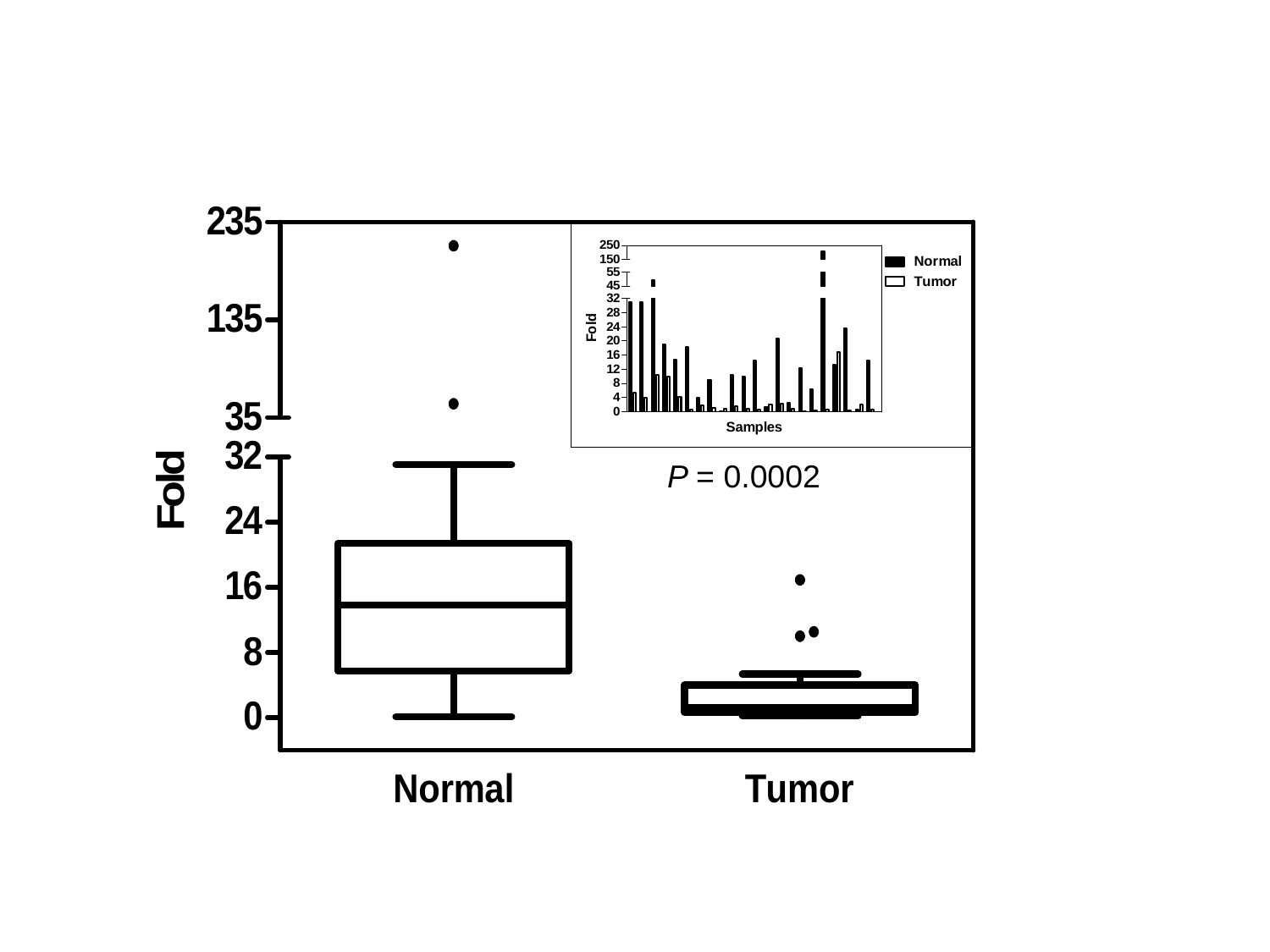

P = 0.0002

Supplement: Additional file 10 — miR-215 expression is decreased in colorectal cancer compared to normal colorectal specimens by real time qRT-PCR analysis. Expression level of miR-215 was normalized by the internal control RNU6B in each sample. P = 0.0002, two-tailed paired Wilcoxon test. [file 1476-4598-9-96-S10.PPT]
