# Supplementary material for: Molecular mechanism of chemoresistance by miR-215 in osteosarcoma and colon cancer cells
Source: Mol Cancer. 2010 Apr 30;9:96. doi: 10.1186/1476-4598-9-96 (PMC2881118; doi:10.1186/1476-4598-9-96)
Supplement: Additional file 8 — Antagonizing miR-215 by LNA anti-miR reverses the impact of miR-215 on the cell cycle. (A) Knock-down miR-215 decreased G2 phase and increased S phase of the cell cycle in HCT 116 (wt-p53) cells, no such effects were found in HCT 116 (null-p53) cells. HCT 116 (wt-p53) cells and HCT 116 (null-p53) cells were transfected with 100 nM miR-215 for 24 h. Cell cycle analysis was performed after transfected with 100 nM LNA-miR215 for 48 h. (B) In parallel, LNA-miR215 prevented the induction of p53 and p21 expression in HCT 116 (wt-p53) cells analyzed by Western immunoblot analysis. LNA-control was the negative control. This experiment was repeated two separate times, and similar results were obtained. The representative flow cytometry pattern was shown. [file 1476-4598-9-96-S8.PPT]

## Slide 1
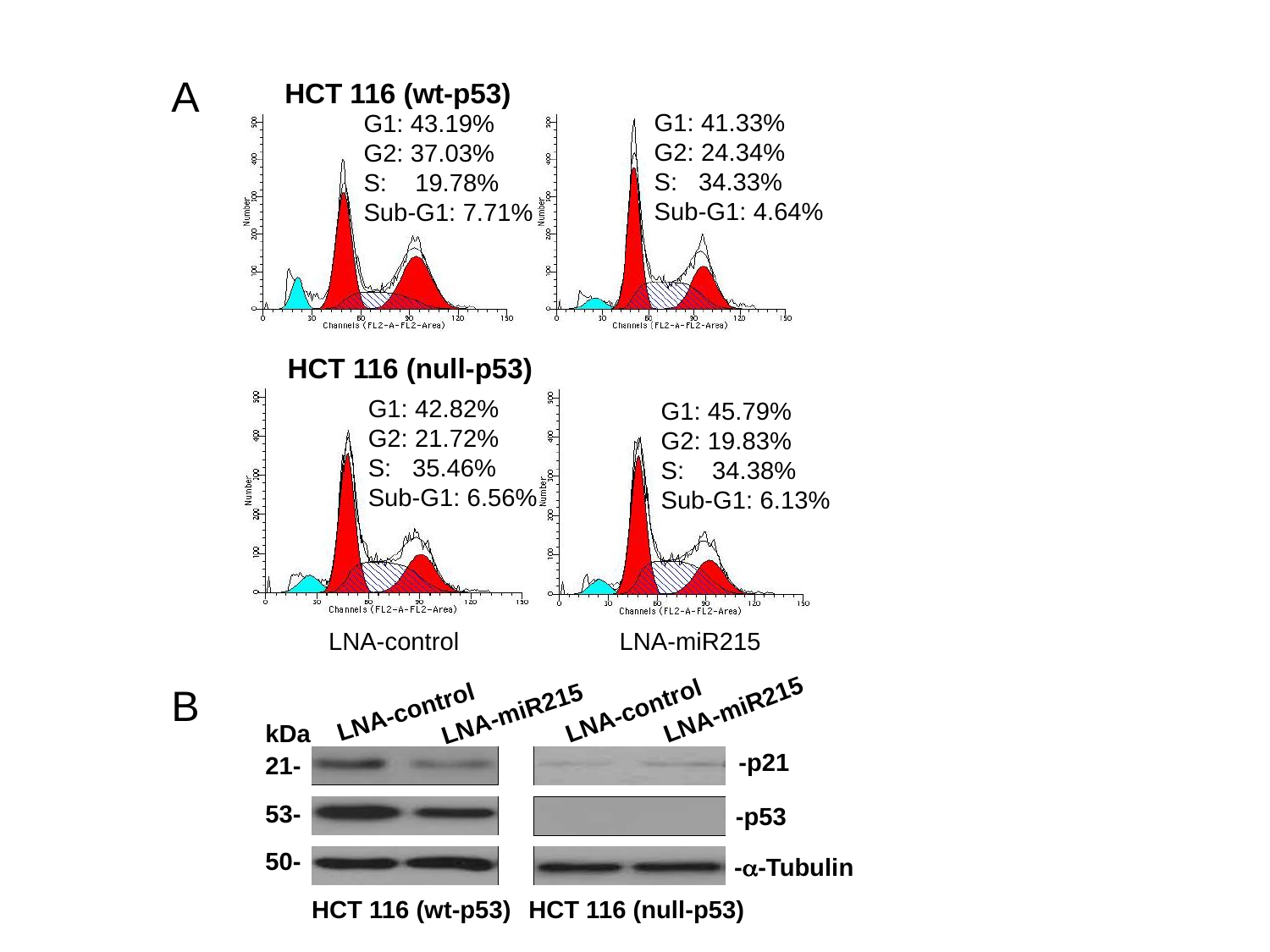

A
B
HCT 116 (wt-p53)
G1: 41.33%
G2: 24.34%
S: 34.33%
Sub-G1: 4.64%
G1: 43.19%
G2: 37.03%
S: 19.78%
Sub-G1: 7.71%
HCT 116 (null-p53)
G1: 42.82%
G2: 21.72%
S: 35.46%
Sub-G1: 6.56%
LNA-control LNA-miR215
G1: 45.79%
G2: 19.83%
S: 34.38%
Sub-G1: 6.13%
LNA-miR215
LNA-control
LNA-control
LNA-miR215
kDa
-p21
21-
53-
50-
-p53
-α-Tubulin
HCT 116 (wt-p53)
HCT 116 (null-p53)
