# Supplementary material for: Molecular mechanism of chemoresistance by miR-215 in osteosarcoma and colon cancer cells
Source: Mol Cancer. 2010 Apr 30;9:96. doi: 10.1186/1476-4598-9-96 (PMC2881118; doi:10.1186/1476-4598-9-96)
Supplement: Additional file 9 — The expression of miR-215 in human colon cancer stem cells is elevated. (A) FACS analysis was performed to sort colon cancer stem cells using CD133 and CD44 as the markers. CD133+HI/CD44+HI cells were considered as the colon cancer stem cells. CD133+/CD44+ and CD133NEG/CD44NEG were considered as the colon cancer cells. (B) Expression of miR-215 in human colon cancer stem cells was analyzed by real-time qRT-PCR. The value of miR-215 in the CD133+/CD44+ colon cancer cells was set at 1, the relative amount in CD133+HI/CD44+HI colon cancer stem cells and CD133NEG/CD44NEG colon cancer cells was showed as the fold induction. (C) The expression of DHFR and TS proteins was decreased in CD133+HI/CD44+HI colon cancer stem cells compared to control cell population analyzed by Western immunoblot analysis. [file 1476-4598-9-96-S9.PPT]

## Slide 1
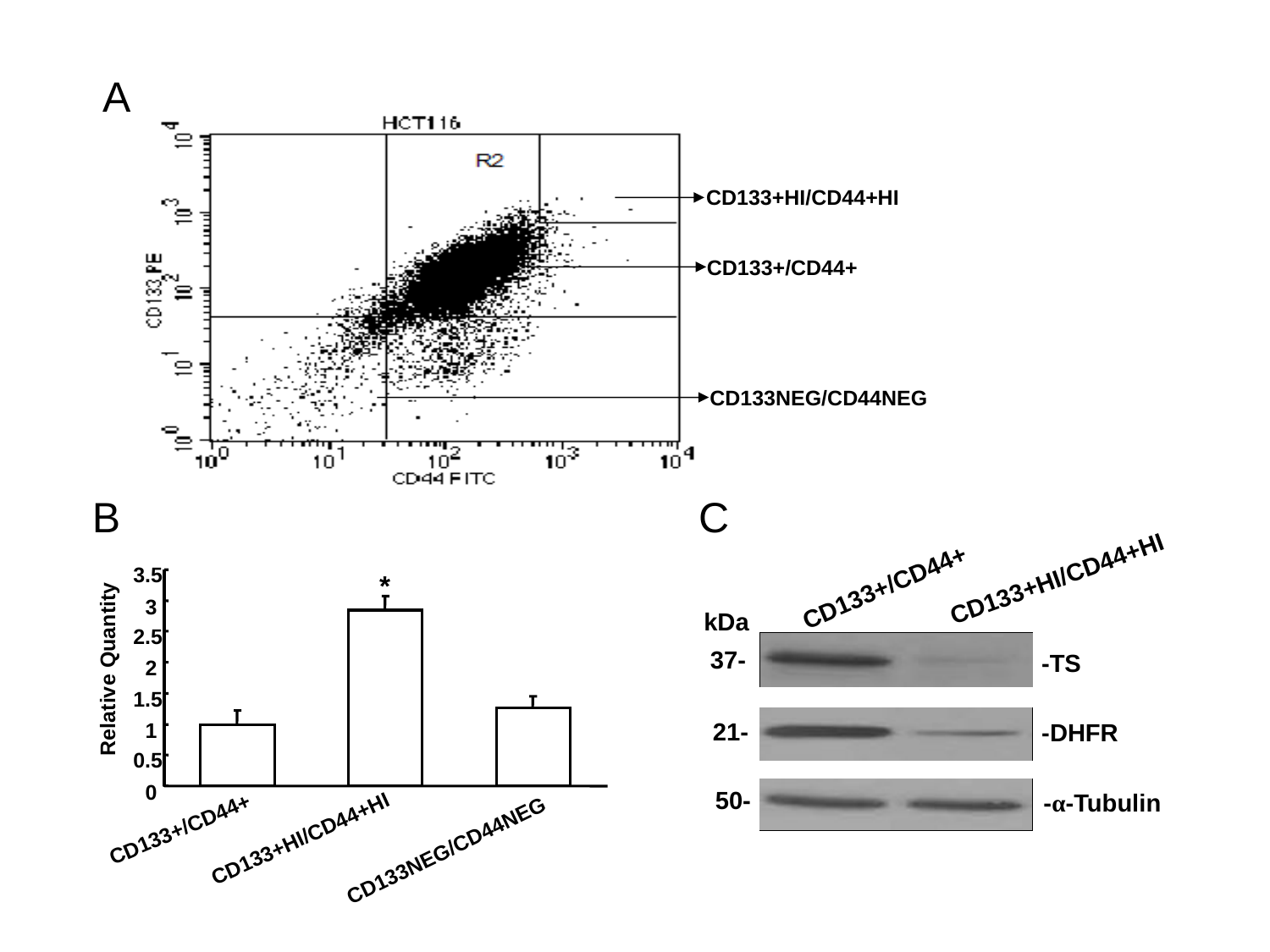

A
CD133+HI/CD44+HI
CD133+/CD44+
CD133NEG/CD44NEG
B C
CD133+HI/CD44+HI
CD133+/CD44+
kDa
37-
21-
50-
-TS
-DHFR
-α-Tubulin
3.5
*
3
2.5
2
1.5
1
0.5
0
Relative Quantity
CD133+/CD44+
CD133+HI/CD44+HI
CD133NEG/CD44NEG
